# How Enaction and Ecological Approaches Can Contribute to Sports and Skill Learning

**DOI:** 10.3389/fpsyg.2020.523691

**Published:** 2020-10-23

**Authors:** Carlos Avilés, José A. Navia, Luis-Miguel Ruiz-Pérez, Jorge A. Zapatero-Ayuso

**Affiliations:** ^1^Department of Languages, Arts and Physical Education, Faculty of Education, Complutense University of Madrid, Madrid, Spain; ^2^Departamento de Ciencias Sociales, de la Actividad Física y del Ocio, Facultad de Ciencias de la Actividad Física y del Deporte, Universidad Politécnica de Madrid, Madrid, Spain

**Keywords:** embodied cognition, acquisition, mastery, expertise, physical education

## Abstract

The purpose of this paper is to explain learning in sports and physical education (PE) from the perspective of enactive and ecological psychology. The learning process is first presented from the enactive perspective, and some relevant notions such as sense-making and sensorimotor schemes are developed. Then, natural learning environments are described, and their importance in the human development process is explained. This is followed by a section devoted to the learner’s experience in which some research methods are explained, such as neurophenomenology, in addition to self-confrontation, interviews aimed at bringing out the meaning, sensations, and emotions that performers experience when they are immersed in their sport or a PE class. The sections on the ecological approach deal with the attunement, calibration, the education of intention, and the importance of representative experimental designs. The last section addresses the main similarities and differences between the two approaches. Finally, we state our theoretical position in favor of a common project that brings together the main elements of both post-cognitive approaches.

## Introduction

There has recently been an increase in the number of papers published linking enactivism and ecological psychology. This is evidence of the scientific community’s growing interest in these methodological concepts and proposals (e.g., Segundo-Ortin, 2020). This trend encourages us to envisage a confluence between these embodied approaches in today’s post-cognitive era (Lobo, 2019). Classical cognitivism and information-processing theory based on a computer-based analogy of the mind, have both been strongly criticized. It currently appears very difficult to accept, given an analysis of the athletes who are active in such a rapidly changing environment as is the sporting world, the explanation that learners use representations of the world, motor programs, and rules to act (Moe, 2005; Breivik, 2007).^1^ A revised paradigm of the mind based on this critique arose some years ago wherein mental processes do not occur solely in the head of the performer. The approach corresponds to the “4E” theory on cognition as embodied, embedded, enacted, and extended (Rowlands, 2010).^2^

According to Gallagher (2017), these four pillars of post-cognitivism have been redefined over time, and some researchers have added more “Es” (ecological, empathic, experiential) and even an “A” (4E&A, affective) to the original idea (see also Menary, 2010; Dotov, 2016; Higueras-Herbada et al., 2019). Gonzalez-Grandón and Froese (2018) have developed an excellent explanation of the four “Es,” which can be summarized as follows: *embodied* means that bodily structures constitute the learner’s cognition, “the bodily realization of cognitive abilities as constitutive for their achievement” (p. 190); *embedded* means that the learner’s cognition is situated in an environment and within a specific context that offers affordances by which to act; *enacted* means that “cognition and consciousness emerge only through the active embodied interaction, or structural coupling, of an autonomous living system with its environment” (p. 190); and *extended* means that “cognition is extended beyond the boundaries, thus being inherently connected with the respective physical or sociocultural environment” (p. 190).

These post-cognitivist approaches to cognition have significant implications that affect our understanding of the process involved in learning sensorimotor skills (Di Paolo et al., 2017) and in sports education (Chow et al., 2016). Performers bring sensorimotor skills to bear in all kinds of learning contexts: in natural situations, such as when children play with their friends or parents in a park close to home; in more structured learning environments, such as that usually found in a PE class at school; and in individual and group sports training sessions. For Dreyfus and Dreyfus (1986), to achieve proficiency or expertise in a particular task or sport, an apprentice must undergo a long process of practice, trial, and error. The expert level is the maximum expression of learning and optimization. In their enactive proposal, Di Paolo et al. (2017) advocate an open-ended and never-ending learning process. Over time, “mastery is the ongoing process by which the agent continuously adapts to the challenges of a changing world” (p. 107). Footballers, skaters, climbers, tennis, and other sports players, constantly adjust their sensorimotor organization to all kinds of changes. These adaptations occur every second, from 1 min to the next, throughout a training session, or even from 1 week to the next.

In this paper, we focus on enactivism and ecological psychology to explain skill acquisition in radical embodied cognitive science, which adopts elements of the ecological approach, dynamical systems theory, and the key notions of the enactivist movement (Chemero, 2009). This theoretical integration will later help us explain the incorporation of non-linear pedagogy in sports teaching and PE. The primary objective of this paper is to show how these approaches can improve our understanding of some aspects of the performer’s acquisition process in sport, PE, and daily activities. This paper will give a broad outline of general ideas that can guide practitioners, learners, and academics. Despite theoretical discrepancies, we are in favor of a common project that brings together both post-cognitive approaches.

The first section of this paper sets out the main notions associated with enactive learning. The second section then presents the core notions of ecological psychology in skill acquisition. The third and final section addresses a number of divergences and connections between these approaches.

## Enactivism in Skill Acquisition

The enactive approach to cognition was first introduced in 1991 by Varela, Thompson, and Rosch in their seminal book *The Embodied Mind*. For these authors “cognition is no longer seen as problem solving on the basis of representations; instead, cognition in its most encompassing sense consists in the enactment or bringing forth of a world by a viable history of structural coupling [*sic*]” (p. 205). This initial research program and its founding ideas established a roadmap that researchers and thinkers from different fields of study have subsequently followed, and which is currently in full evolution.

The approach has its roots in phenomenological philosophy (Gallagher and Zahavi, 2008; Gallagher, 2017), and is closely linked to the evolutionary changes that occur in humans during ontogeny, hence its relevance in explaining learning (Di Paolo, 2019). Enactivism is a non-representational approach that adopts world-involving explanations: “cognitive activity is co-constituted by agent and world” (Di Paolo et al., 2018, p. 333). Some ideas are closely related and are essential to an understanding of enactivism: autonomy, sense-making, emergence, embodiment, and experience (Di Paolo et al., 2010). An organism, and in the case that concerns us here, a *learner* of a certain sport is an autonomous system. According to Thompson (2007) “a distinctive feature of the enactive approach is the emphasis it gives to autonomy. In brief, an autonomous system is a self-determining system, as distinguished from a system determining from the outside, or a heteronomous system” (p. 37). For Thompson and Stapleton (2009) “the enactive approach starts from the question of how a system must be organized in order to be an autonomous system—one that generates and sustains its own activity and thereby enacts or brings forth its own cognitive domain” (pp. 23–24).

Another crucial notion is sense-making, meaning “creation and appreciation of meaning,” which arises in the agent’s interactions with the world (Di Paolo et al., 2010, p. 39). If we consider an agent playing a sport, “significance and valence do no pre-exist ‘out there,’ but are enacted” (Thompson, 2007, p. 158), or in the words of Gallagher (2017), “the world (meaning, intentionality) is not pre-given or predefined, but is structured by cognition and action” (p. 6). The agent, through an active sensorimotor engagement with his or her activity, transforms the world “into a place of salience, meaning, and value — into an environment *(Umwelt)* in the proper biological sense of the term. This transformation of the world into an environment happens through the organism’s sense-making activity” (Thompson and Stapleton, 2009, p. 25). “Sense-making is the interactional and relational side of autonomy. An autonomous system produces and sustains its own identity in precarious conditions and thereby establishes a perspective from which interactions with the world acquire a normative status” (p. 25). When an athlete is actively committed to his or her activity, some elements or objects become more relevant than others. According to Di Paolo et al. (2017):

Sense-making does not imply sophisticated kinds of cognition, but it is implied in them. It is what is common to basic minds (Hutto and Myin, 2013) and human minds. To be clear, by “sense-making,” then, we refer to the notion that objects or events become meaningful for an agent if they are involved in the normatively guided regulation of the agent’s activity (e.g., by triggering or mediating it). This mode of relating to the world is the active making sense of a situation and the orientation of the agent toward a course of action that is adequate to it. (p. 123).

### How We Learn to Act and Perceive

Sensorimotor learning involves changes that occur in the behavior of the agent. These changes are non-linear and are constrained by the interaction of a large number of factors, such as the learner’s motivation to acquire a new skill, their sensorimotor coordination during the learning process, the number and variety of opportunities available to them to practice, and even the sociocultural environment in which they grow, develop and learn. The learner needs these possibilities. Adolph et al. (2012) showed that even the most natural or ontogenetic skills such as walking require immense amounts of practice. In a natural environment of free play, toddlers aged between 12 and 19 months of age walk an average of 2,368 steps an hour. The next milestone is to achieve sensorimotor mastery that will allow the toddler to walk very fast or run around without falling. Movements are fundamental since motor actions generate new opportunities for learning or *cascades of development* in domains that go beyond the mere sensorimotor (e.g., intelligence). In response to this, Adolph and Hoch (2019) highlight the embodied, embedded, enculturated, and enabling nature of human movements.

Enactive learning is eminently non-representational, evolutionary, and dynamic. Learners are the product of their history of sensorimotor coupling with the environment, depending on their phylogenesis, ontogenesis, and cultural setting (Varela et al., 1991). To paraphrase Antonio Machado’s famous poem, so significant to enactivists, there is no fixed path or pre-given world that guides our way forward (Thompson, 2007, p. 13). Sensorimotor couplings correspond to earlier, ongoing interactions that leave traces or create habits, the path of learning is the product of creative interactions between learners and their environment. For Hutto and Myin (2013), the arguments that explain the changes in the agent can be found in the developmental-explanatory thesis, “which holds that mentality-constituting interactions are grounded in, shaped by, and explained by nothing more, or other, than the history of an organism’s previous interactions. Sentience and sapience emerge through repeated processes of organismic engagement with environmental offerings” (p. 8).

From the enactive point of view, the learner’s active movements have a relevant role in the emergence of cognitive and learning processes and perceptual learning (Bermejo et al., 2020). Doing and learning by doing is fundamental in couplings between the environment, the brain, and the body of the performer (Gallagher, 2017). Enactivists explain ontogenetic changes and sensorimotor development “as the growth of a network of stable patterns and the relations between them” (Di Paolo et al., 2017, p. 161). Through practice and experience, the performer will expand his or her sensorimotor repertoire and will reach a higher level of dexterity and mastery. Di Paolo et al. (2017) define it as follows: “Mastery is the ongoing process by which an agent continuously adapts to the challenges of a changing world. In our proposal, mastery consists in the refining and acquiring of new sensorimotor responses and their integration into an existing repertoire” (p. 37).

Di Paolo et al. (2017) explain the process of enactive learning through a dynamical system interpretation of Piaget’s theory of equilibration (p. 88). In short, learning involves going through the phases of assimilation, accommodation, and equilibration. According to these authors, the performer acquires sensorimotor schemes with practice. A sensorimotor (SM) scheme is “an organization of SM coordination patterns” (p. 90). In turn, a scheme involves a whole sequence of coordination patterns. If we think of a child who is learning to ride around a circuit on a bicycle with training wheels, the sequence would involve the following patterns: keep the handlebar in the correct position to go straight, pedal at a constant rate, look forward to know when to turn, pedal slower and move the handlebar slightly to the left to turn left, and so on. In this case, for the performer, *assimilation* consists of trying to maintain the stability condition even when variations arise that may affect him or her, such as performing the same circuit just after a rain shower. *Accommodation* is “plastic change that re-establish[es] a scheme” (p. 91), how to perform the same task, but this time without training wheels. Finally, *equilibration* is the last phase of an open, permanent, and endless process of learning. Here, the performer adapts to a variety of practice conditions “aimed at maximizing the stability of each scheme against violations of the transition and stability conditions resulting from environmental perturbations or internal tensions” (p. 91). Practical examples of this last phase are using lighter and heavier bicycles and riding around the same circuit but with steeper and flatter slopes, or on a variety of surfaces, such as dirt, asphalt, tile, etc.

### Natural Learning Environments

Not all learning takes place in formal settings with purposeful teaching programs such as those implemented in schools and sports clubs. A great deal occurs naturally. Stewart (2010) emphasizes the importance to the learner’s autonomy of action and the learning that takes place in or near the learner’s current stage of development, and criticizes the Shanonian notion of information and instructional teaching processes:

“Learning” can only be a modification of the developmental process; this means that what can be “learned” is both enabled and constrained by the epigenetic landscape. Development, and therefore learning, is essentially an endogenously self-generating process; it is, therefore, unnecessary—and impossible—to “instruct” it from the outside. This runs directly counter to the widespread notion that “learning” is a process of “instruction,” by which is meant a process of information transfer from teacher to pupil (pp. 8–9).

Gallagher (2017) mentions the importance of *natural pedagogy* in a child’s learning. The process of upbringing, non-formal teaching, and interaction with others (i.e., intersubjective education) determines the amount of attention we pay to some objects or events over others. A natural context in which enactive intelligence manifests itself is found among populations who depend on the sea for their livelihood. These groups, called *Sea Nomads*, include the Moken and Orant Laut of Malaysia and Indonesia and the Bajau Laut of the Philippines, Malaysia, and Brunei. These villagers spend an average of six or even 10 h a day in the water, and half of this time is spent underwater. The sea is the children’s playground, and the adults’ workplace (Abrahamson and Schagatay, 2014). Their enactive-aquatic intelligence is embodied and enacts with objects and situations since it allows them to adapt to the problems posed by their aquatic environment. Their coupling is such that their children can see perfectly well underwater without the help of goggles, a finding that was of great interest to researchers (Gislén and Gislén, 2004). This lifestyle has led to major adaptations similar to those found in many marine animals, namely, their diving reflex and their clarity of vision underwater. Gislén et al. (2003, 2006) wondered whether the visual acuity of these groups was genetic or the result of an extensive history of coupling and co-dependence of these children with water. After carrying out different studies, they concluded that the superiority of these Moken or Bajú children with respect to European children was a consequence of a long evolutionary history of co-determinations with the marine environment (Gislén et al., 2003). For these researchers, spending their lives in the water has taught these children to constrict their pupils so that they can see clearly when they are submerged. Thousands of years of structural coupling with water has facilitated the development of the underwater sensorimotor skills they need to survive. When these individuals dive into the water, their intelligence extends beyond their hands and is distributed in the fishing utensils they use, and they demonstrate a refined sensorimotor skill that allows them to move freely in the marine environment, making their aquatic experiences much more than mere acts of sensorimotor coordination.

### The Subjective Universe of Learners and Experience

The subjective universe is independent of any external analyzing and quantifying observer. No external observer can see what the learner, practitioner or team sees, feels, and lives from their point of view, with their biological idiosyncrasies, sensorimotor skills, knowledge, psychological characteristics, and their experience during the activity. Agents themselves interpret the usefulness of their actions. It is their subjective world (*Umwelt*) that emerges in these situations (Von Uexkül, 1951) − a world of interactions and co-determination with different levels of analysis and organizational domains, in which the specific motivations and intentions of the agents arise during the action. Von Uexkül proposed the concept of *Umwelt* to highlight the specific relationships between agents and their environments. They always perceive the world from their point of view. As Merleau-Ponty (1985) explains, the individual is not only a body, not only a physical structure but also a being that lives and experiences, that manifests an external and internal dimension when relating to its environment. From an enactivist perspective, learners are beings in a situation where they have an intense relationship with their environment, in which their subjective world is intensely involved, and is absorbed in their actions.

Educational contexts are laden with embodied situations of acquisition and sensorimotor knowledge. Although the school system insists on eliminating the body from education, learners must be present with their body in the world. Learners are thus open to possibilities, operating bodily within, and react to the specific situations in which they find themselves. They enact to obtain the information they need from the environment and make decisions without the need for complex cognitive operations or mental representations (Button et al., 2012; Avilés et al., 2014; Davids et al., 2015). The unit of analysis is no longer the isolated individual, but rather the system made up of that individual *in situ*, in co-dependent and dynamic interaction with the environment in which emerging, self-organization processes occur (Varela et al., 1991). Learners regard themselves as individuals in action and in a situation, in a dialectical relationship with their surroundings and with the objects around them.

One of the crucial challenges and novelties of enactivism at the methodological level is to articulate descriptions of learners’ experiences using objective behavioral data. Varela (1996) called this approach neurophenomenology − the way of studying first-person subjective experience and third-person objectification. The key is to create a fruitful circularity between phenomenology and cognitive science. One of the characteristics of this method is that both participants and investigators need to be properly trained to use it correctly. A second person, i.e., an investigator or *empathic resonator*, is sometimes called in to act as mediator or coach. Their role is to be aware of the signs and indicators of the study participant (i.e., the first person) in order to interpret the data they express, such as phrases, body language, and expressions, etc. (Depraz et al., 2003).

The capacity to access learners’ consciousness is a fundamental challenge for investigators. The objective is to bring out the significant elements of the experience when practicing or learning a sports activity. There is an important body of literature in which researchers often combine biomechanical data with that obtained from interviews with athletes and learners in PE and sports (Hauw and Durand, 2007; Bourbousson et al., 2012; Sève et al., 2013; Evin et al., 2014; Terré et al., 2016; Rochat et al., 2017, 2019; Hauw, 2018; Récopé et al., 2019). Although each study is unique, the general method has been to reconstruct the *course of action* (Theureau, 2010) to “capture” the performer’s experience by verbalizing it in self-confrontation interviews. During the interviews, the researchers present the performers with videos and biomechanical data that allow them to relive the experience and more easily unleash the feelings, emotions, concerns, etc. In their study in rowing, Sève et al. (2013) analyzed athletes’ subjective perception of the synchronization of their movements, which are not very noticeable externally. Reconstruction and access to the athletes’ *course of action* allowed coaches to identify temporary movement dysfunction. It is important to detect this mismatch to readjust the biomechanics of movements in training.

It is essential to immerse ourselves in or break down the moment of learning from the point of view of the learner in PE, because, as Masciotra et al. (2008) explain, learning occurs from the perspective or optic of the learner, and he or she will have significant access to new skills when this converges with emotions, feelings, beliefs, previous experiences, etc. that are brought into play in the situated action. This has two very important consequences in PE. On the one hand, it optimizes the diagnostic assessment of the student and the initial knowledge of each student. On the other hand, it establishes a reactive, empathetic, skillful, and recurrent assessment that obtains information from the student’s perspective and, simultaneously, gives empathic feedback that enhances the meaningfulness of the activity experienced by the learner in the context of the PE class. The world of teacher-student interaction during PE lessons appears to be mediated by intersubjective perspectives that show that teacher empathy is very relevant to motor learning.

### The Role of Practitioners in Developing Skill Mastery

As mentioned above in the context of learning or mastery, enactivism relies on the tools of dynamic systems theory. Like ecological psychology, both approaches view the learner as a complex adaptive system that in turn forms a system with the environment. The self-organization of learner behavior is a crucial element in the autonomy of action. Several years ago, an applied proposal called the Constraints-Led-Approach (CLA) emerged in the field of sports science and PE. This explains the emergence of a new pattern of coordination that involves the interaction between constraints associated with the task, the environment, and the learner (Renshaw et al., 2019). CLA is based on the principle that the learning process does not follow a linear trajectory and therefore results in a non-linear pedagogy (Chow et al., 2016). In this regard, for Davids et al. (2008) non-linear pedagogy is:

A theoretical foundation that views learning systems as non-linear dynamical systems. It advocates that the observed properties of dynamical human movement systems form the basis of a principled pedagogical framework. In particular, non-linear pedagogy advocates the manipulations of key constraints on learners during practice (p. 224).

If we accept that perception, coordination and cognition can be explained through the self-organization of behavior, we must believe that non-linear pedagogy can be related to enactivism. In an enactive pedagogy, the practitioner is always present to promote learning, but the question is, how? Although practitioners might adopt a traditional approach and teaching method, they must above all design learning environments that favor a varied landscape of affordances. In these scenarios, the practitioner’s mission is to become a true “environment architect” insofar as sensorimotor exploration and autonomous discovery of solutions will be accompanied by more selective and less frequent use of verbal information (Renshaw et al., 2019). Therefore, teaching sessions involving children from 2 to 6 years of age should invite them to explore and to allow their sensorimotor behaviors to emerge spontaneously (Équipe des Conseillers Pédagogiques en EPS du Bas-Rhin, 2015). Returning to the issue of autonomy of action, a study published by Récopé et al. (2019) found that certain professional volleyball players strayed from the established game system: “It should also contribute to explain why some people (here some players) have some difficulties to follow the prescription, including the role distribution in a collective organization (here the game system)” (p. 236).

To make it easier for the reader to understand the acquisition process, we will take the example of tennis. There is no doubt that an apprentice aspiring to be a good tennis player will need thousands and thousands of practice shots to reach a good level of play. However, since tennis is not a predefined world, each shot or movement is different. In tennis, one of the most interesting moments of the game is the return of serve, probably due to the receiving player’s impressive responses to a ball traveling at more than 200 km/h. One of the movements that tennis players acquire after years of practice is the split-step. This is a hop-jump sequence that the receiver performs by jumping or taking off from the ground just before the server hits the ball. Until recently, tennis players were thought to have directional anticipatory behavior, that is, they were able to anticipate the server’s movements and move and jump to the side where the ball would land in order to respond before the server hit the ball. However, recent research has shown that expert tennis players follow a *neutral* jump pattern, i.e., they do not move to either side during the split-step (cf. Avilés et al., 2019).

From the enactive perspective, studying the split-step gives insight into how tennis players with different levels of expertise function. Firstly, beginners and basic level players cannot do the split-step; they must learn it naturally through sensorimotor adaptation during practice. There is no way of knowing exactly when they will learn it or when this movement will first emerge *naturally* during the game. Secondly, non-anticipatory neutral behavior indicates that the expert receiver creates meaning or *sense-making* in each serve and return sequence, and even that considering their intentions, emotions, and movements, a participatory sense-making of the interactions between both players will emerge (Di Paolo et al., 2010). Thirdly, several scholars criticize excessive intellectualism and argue that mental representations are not needed to act competently (Noë, 2009). In fact, in the case of a tennis ace, these images or representations could impair their performance. The body-mind unit evolves, and this is reflected in the mastery the player brings to the sport. As Varela et al. (1991) put it: “As one practices, the connection between intention and act become closer [*sic*], until eventually the feeling of difference between them is almost entirely gone” (p. 29).

For the embodied and enactive approaches, being skilled means acting intelligently in a situation in which the individual is both *situating* and *situated* (Masciotra et al., 2008), and in which he or she establishes a dynamic and adaptable relationship. Acting skillfully means using enactive intelligence, to the extent that it activates the individual’s adaptive capacity as a learner, showing control over themselves and the situations around them (Noë, 2004). In this adaptive process, cognition is distributed throughout the body, the learner does not operate outside the world, rather, it is the interaction of the athlete with his or her world that gives meaning to learning and performance. These enactions take place in natural and formal educational contexts.

Returning to enactive ideas, action spaces become a network of relationships, which are embodied between the agent (practitioner and/or learner) and the environment. Training, according to Stewart et al. (2010), becomes a conscious experience of the experience of acting, where the performers as “cognitive systems are always engaged in contexts of action that require fast selection of relevant information and constant sensorimotor exchange” (p. x). Learning skills means effectively and efficiently changing the knowledge of the acting agent, changing their sensorimotor patterns, their way of acting, the meanings of their actions, and their intentions. It involves creating an enacted and embodied itinerary in which the agents progress from incompetence to expertise.

In enactivism, motor learning involves refining adaptation processes through a history of structural couplings with the environment. A situation is a specific space-time that influences the way individuals act. For enactivists, the practitioner needs to create situations that favor adaptation processes in which athletes co-determine with their environment, and which facilitate the emergence of the appropriate situation-specific motor patterns. As a result of various couplings, motor skills *emerge* more than they are *acquired* (van der Kamp et al., 2019). The Fosbury flop high jump does not exist in itself, it only exists when the athlete enacts with the situation and clears the bar. As Merleau-Ponty (1985) indicates, the individual is inseparable from the environment in which he acts. Sports action exists to the extent that athletes are in a position to act, and is defined, in this case, by their motor coordination and their sensitivity to changes in the physical and material environment in which they act (McGann et al., 2013).

One of the questions posed by researchers is whether mental representations are needed in these intense relationships between athletes and their sports environment − whether it is appropriate to claim the existence of such internal constructs, or whether it is direct experience, the athletes’ direct contact with their environments, that drives the emergence of the sensorimotor patterns of solution. Practitioners face the challenge of designing and promoting situations that favor enaction. Understanding the situations in which learners act in PE classes involves analyzing the interactions and couplings that favor the emergence of significant sensorimotor patterns to solve the problems that arise. It is important to examine these perception-action cycles and the dynamic interactions they elicit between the brain, the body, objects, materials, people, and the context in general. In the field of sports, the questions raised for researchers are: how do athletes interact with their environments? What favors or hinders these co-determinations or couplings with the environment? Or, how do sensorimotor patterns of action emerge in these co-dependence processes?

Furthermore, and along the same lines, it is important for the practitioner to calibrate the degree of variability in daily practice. Renshaw and Chow (2018) maintain that practitioners must take variability into account to promote learning:

The amount of variability designed-in to a session needs to be matched to the learner. For the beginner level player, low task and environmental variability may be beneficial to guide exploration toward one or two functional solutions. In contrast, the more expert performer may be presented with greater variability in individual, task and environmental constraints to promote more adaptable behavior. Knowledge of ‘critical values’ (i.e., the amount of variability that will lead to instability and the search for new solutions) is important for practitioners and needs careful management and awareness of the implications for placing individuals in these critical (‘red’) zones (p. 12).

## Skill Acquisition From the Ecological Perspective

Ecological psychology was first formulated by the psychologist James J. Gibson (1979/1986) in opposition to the (by then prevailing) cognitivist approach. The ecological approach to visual perception was originally conceived to explain how animals control movement in their environment. Subsequent researchers have made significant contributions to the development of a theory founded on motor control and learning in humans (Michaels and Carello, 1981; Turvey, 1992; Withagen, 2004; Jacobs and Michaels, 2007) and its application in sport (Araújo et al., 2006; van der Kamp et al., 2008; Fajen et al., 2009). In this section, we will try to describe the advances made in the ecological approach to sport expertise and skill acquisition.

The ecological approach championed direct perception, which involves some core considerations (Gibson, 1979/1986). First, that information is rich enough to produce perception, and no computations or inferences (i.e., knowledge stored in the memory) are required to perceive the energy patterns in the ambient array. Second, that the available information specifies environmental properties that in turn offer invitations to act. Affordances can be conceived as the opportunities for action that the athlete perceives from informational variables emerging from the environmental specifications. Third, perception and action are coupled processes that mutually influence each other. How an athlete becomes an expert, or how a learner is capable of acquiring new skills, has been explained by three interconnected stages: education of attention, calibration, and education of intention (Jacobs and Michaels, 2007).

### Education of Attention or Attunement

When the perception of a property involves one-to-one mapping with respect to environmental energy patterns (1:1), then that informational variable is *specific* to that property. For instance, the variable tau (τ) under certain circumstances signifies the ratio expansion of an incoming object (e.g., a ball in a penalty kick), which in turn specifies the time-to-contact (Savelsbergh et al., 1991). However, performers detect (and use) some informational variables that may not perfectly correlate with environmental properties but can still be useful. These are the non-specifying variables (Withagen and Chemero, 2009). As one might observe, the usefulness of variables to accurately control movement depends on the reliability of the information (degree of specificity of the variable). For example, during a penalty kick, the goalkeeper may rely on non-specific variables, such as the penalty-taker’s direction of gaze during the run-up (which does not necessarily match the direction of the kick; Wood and Wilson, 2010), or they might base their dive on other, more reliable, variables observed closer to ball contact, such as the orientation of the non-kicking foot (Lopes et al., 2014). In penalty-saving, informational variables that unequivocally determine the trajectory and the speed of the kick are extracted from the ball’s flight.

The education of attention is the convergence from the least to the most (1:1) specifying variables. With practice, performers learn to rely on more useful variables to control a particular action. A recent study applied this theoretical concept to penalty kicks, with promising results (Dicks et al., 2017). Goalkeepers improved their rate of successful saves after on-field training. During training sessions, they were forced to pick up information closer to the point of contact with the ball by placing three potential penalty-takers who simultaneously started the run-up to the ball, but only one kicked the ball. In other words, the goalkeeper learned to become attuned to more specific informational variables during the penalty kick.

### Calibration

There is an ample body of research (cf. van Andel et al., 2017) showing that the perception of opportunities for action (affordances) are scaled to the performer’s ability, such as their size (e.g., Warren, 1984) and personal capabilities in terms of action (e.g., Dicks et al., 2010b). Fajen’s affordance-based control approach establishes that an athlete’s action capabilities regulate their own and other’s (opponents) affordance perception, creating a boundary between achievable and non-achievable actions (Fajen, 2005; Fajen et al., 2009). In other words, successful control of movement is predicated on the basis of the relationship between the maximum capabilities of action and the space-time constraints of the task.

Continuing with the penalty kick example, one goalkeeper may be attuned to the most specific information about the future location of the ball: the trajectory of the ball during the first moments of flight. However, that information is typically picked up too late, leaving the goalkeeper insufficient time to complete the dive with guarantees (i.e., arrive at the right time). Therefore, if goalkeepers do not calibrate their agility to the expected demands of the situation by waiting for the most reliable information, they will move to the same side as the ball (the right place), but too late to intercept the ball (Navia et al., 2017). Hence, the space-time constraints of the task (speed of the ball, distance traveled) would need to be calibrated for maximum agility (speed of movement) if they are to achieve their objective of stopping the ball (see a detailed model of this in van der Kamp et al., 2018). Studies suggest that goalkeepers scale their timing of the save to their capabilities (Dicks et al., 2010b); more agile goalkeepers start the saving action later (closer to ball contact) and less agile ones dive earlier. Interestingly, more agile goalkeepers were found to save more penalties (Dicks et al., 2010b).

### Education of Intention

Education of intention is defined as the selection of affordances that guide behaviors. In other words, it is about decision-making during an action. The selection of action is related to the perception of affordances, which in turn depends on scaling actions (calibration). For example, there are some basic scenarios in which the athlete’s decision-making takes into account lateral movements (e.g., penalty kick, return of tennis serve). In the case of a penalty kick, the control of the action – where to dive and how to time the dive – would be primarily predicated on the affordance-based control of that interacting situation (see van der Kamp et al., 2018). In Dicks et al. (2010b), more agile goalkeepers who initiated the saving action later saved more penalties than their slower counterparts. The authors suggest that waiting longer allowed goalkeepers to pick up more reliable information and control their actions based on more specifying variables (Lopes et al., 2014). Similar findings have been reported in tennis (Triolet et al., 2013) when, under more lenient space-time constraints, players waited longer, which in turn allowed them to base their action on more reliable information (see also Navia et al., 2018).

However, there are multiple situations where different affordances can be used to guide actions (e.g., imagine a football midfielder just after receiving the ball). Here, ecological dynamics provides a theoretical framework for explaining behavior trends (Araújo et al., 2018). On the premise that behavior emerges from the interaction between the athlete’s characteristics (abilities) and the space-time constraints of the environment, affordance selection is understood as the shift from action modes (e.g., moving forward with the ball, dribbling, passing to a teammate, etc.). These changes between modes of action fulfill a functional criterion. Athletes follow a particular (and stable) action mode until the instability of emerging agent-environment constraints compels them to shift toward another mode during the action. Underlying agent-environment system factors such as the distance between encounters (Esteves et al., 2011; Vilar et al., 2012) have been found to shape changes of action modes and successful performance in sports such as basketball (Esteves et al., 2011), futsal (Vilar et al., 2012), boxing (Hristovski et al., 2006), etc. With practice, performers learn how to become attuned and calibrated to the landscape of affordances to maximize action selection and transition from one action mode to another (Araújo et al., 2019). In this regard, Rietveld and Kiverstein (2014) argue that an animal in a particular *life form* perceives affordances in relation to motor abilities. With practice and experience, as the performer becomes more skillful his or her landscape of affordances becomes richer and more varied. Therefore, two performers at different sporting levels who share the same sociocultural practice could have more relevant or less relevant affordances. This means that for an athlete, affordances are modified and actualized in accordance with the learning process. In the words of Heras-Escribano (2019a): “the action–perception loop changes and it allows us to open new possibilities that were not present before” (p. 87).

Despite these recent contributions, this aspect is still the least developed area within direct learning (Jacobs and Michaels, 2007). In particular, how information from different sources interacts and is integrated remains unsolved. In this regard, the probabilistic functionalism derived from the Brunswik lens model may provide a promising sports framework to further explore the interaction between imperfect information coming from different time-scales: proximal vs. distal (Pinder et al., 2013). For instance, in the football penalty kick, goalkeepers in experiments modified their behavior (timing and side success) whenever situational information concerning the kicker’s preferences was conveyed (Navia et al., 2013). Similarly, goalkeepers show an unusual tendency to dive to either side, regardless of the kicker’s kinematics or record, due to possible negative social judgments if they do not (Bar-Eli et al., 2007).

### Representative Experimental Designs

A core concern in ecological psychology has been the degree of fidelity between behavioral agent-environment system properties and the experimental settings used to test expert performance and perceptual (motor) learning. Since Araújo et al. (2007) reintroduced the original Brunswik notion of representative design (Brunswik, 1956), sports scientists have been concerned about the extent to which experimental conditions influence the perception of affordances and the regulation of actions. Accordingly, there has been growing opposition to some methods used to capture the expertise of athletes (e.g., verbal reports, occlusion techniques, video-based training, etc.) that could hamper perception-action coupling at both the basic, neuropsychological (van der Kamp et al., 2008; Mann et al., 2013) level and the applied behavioral level (Travassos et al., 2013).

In other words, if ecological psychologists hold that performed actions change the way the athlete perceives the world, and affordance perception changes the regulation of actions, then separating or altering the natural coupling between perception and action would significantly distort the picture of perceptual attunement, calibration and affordance selection. For example, in the football penalty kick, findings suggest that differences in information pick up among goalkeepers occur as a function of the type of response required (i.e., joystick or verbal vs. actual save) (Dicks et al., 2010a). Therefore, the representativeness of task design in experimental settings should be assessed and ideally be preserved at the highest level to truly recreate the athlete’s skill performance in a competitive context (Avilés et al., 2019) or actual learning conditions (Pinder et al., 2011).

## Differences and Similarities Between Approaches

Discussing skill acquisition from an enactive and ecological perspective, this paper will now present some divergent points that make it difficult for these approaches to converge or work together. Heft (2020) recently analyzed the most relevant books and articles in this regard and identifies three main discrepancies between direct realism and enactivism: *sensations*, the *concept of information*, and an *organism’s boundaries* (see Heft, 2020 for a review). Cummins (2020), meanwhile, also believes convergence to be difficult, and criticizes ecological psychology, saying: “on the experience side of the account, it has nothing to say about phenomenology, experience, emotions, or feelings” (p. 9). Moreover, Cummins says:

The ecological analysis starts by singling out a “behavior” to be characterized, by fixing the organism/animal/agent and the environment of relevance, and it builds its account from there. In so doing, it frequently has the result that much of the explanatory load normally consigned to hidden interiorities and brains is reduced, but not removed. (p. 10)

Since the publication of *The embodied mind* in 1991, the founders of enactivism have criticized Gibson’s approach. Almost 30 years ago, Varela et al. (1991) explicitly expressed their disagreement, especially with “the act of perceiving being direct” (p. 204). For ecological psychology, information is there for the agent to perceive it directly, and this information affects the perceptual process that guides their actions. For enactivists, these invitations to act (affordances) cannot be captured directly by the agent − they can only be detected or rather *enacted* in a co-determination relationship (Scarinzi, 2011). The concept of functional tonality has many similarities with the previously mentioned *equimentality* of Heidegger (2003) or Gibson’s affordance Gibson (1979/1986). Sounds, movements, gestures, objects, people, weather, etc., mean something, establish something that is perceived and *interpreted* by performers and practitioners. Coaches, in their desire to teach and correct skills, situate their subjective universe in relation to the subjective worlds of their athletes. This embodied orientation is reflected in the current understanding of motor learning in school, where the feelings, emotions, and perception of how learners live and feel teaching, has led to the emergence of new 21st century PE teaching methods, focusing on how to teach learners to understand how they learn (Moy et al., 2019) in an environment full of meaning, mediated by a universe of dozens of students.

Another relevant critique of the ecological approach has been the role played by the movement of the perceiver. Enactivists argue that in the Gibsonian approach, agents and performers play a passive role when perceiving, that is, that the act of perceiving was passive rather than active (Varela et al., 1991). We believe that Gibson’s founding idea Gibson (1979/1986) has always been the idea of seeing the agent as an active explorer, and we, therefore, disagree with this enactivist critique. In this regard, Gibson (1969) mentioned the following about perceptive learning: “It is not a passive absorption, but an active process, in the sense of exploring and searching, for perception itself is active” (p. 4). Three decades later, Gibson and Pick (2000) added: “Information about properties and especially about what they afford is actively obtained by exploring, and after a few more months by actively using objects. Exploring objects and discovering how they can be used is the way meanings are learned” (p. 86). It is also true that the experimental paradigms used by both approaches can induce certain differences. Specifically, many enactivist studies have used sensory substitution or deprivation, which compels participants to perform many active movements to perceive, and this, logically, leads to a sense-making that demands a true commitment from the learner (see Bermejo et al., 2020). However, if we consider ecological sports studies, where the performer has access to information from all their sensory modalities, perception can be rapid and active without the need for many repeated or constant movements to elicit meaning. For example, in a football penalty kick, the goalkeeper moves but only has a few milliseconds to perceive the direction of the ball.

An important limitation for the convergence of both post-cognitive approaches is the use of different concepts and vocabulary. This is a problem for researchers who are compelled to use enactive and neo-Gibsonian terms that generate different interpretations. A common language would facilitate understanding among practitioners such as PE teachers and coaches and help them design practice sessions that encourage emergence or self-organization. Heras-Escribano (2019b) argues that some enactivists use the term *affordances* very lightly, with no regard for the ontological and epistemic consequences (p. 207). On the other hand, it is interesting to note how the term affordances has different interpretations in ecological psychology (e.g., Chemero, 2009; Withagen et al., 2012; Rietveld and Kiverstein, 2014).

Despite apparently studying the same phenomenon, when we look more closely, we realize that both approaches explain the relationship between the performer and the environment differently. From an ecological point of view, the environment is *more objective*; enactivists, however, give more importance to the history and *lived experience* of the agent. As McGann (2016) points out: “it is also important to note that experience is continually sensitive to its own history” (p. 313). Enactivism explores this subjective dimension of actions, motivations, needs, and impulses that compel performers to commit to their environment (Di Paolo, 2005). Each person perceives this enactive relationship according to their personal characteristics, their goals, and their life experience. And it is in this area of this subjectivity where objects and situations make sense to the performers, where they acquire a purpose and utility, and where they have a functional tonality, defined by what can be done with them, by their usefulness. This is an aspect that ecological approaches based on Gibson’s theories reject, despite the numerous coincidences that exist between the two paradigms, since although ecological approaches sometimes use the term enaction, they do so as a way of highlighting the active nature of perception, focusing attention on the environment (Stoffregen et al., 2006). For enactivists, the structural coupling between agents and their environment (couplings) must be regarded as sensorimotor patterns that enable them to carry out actions guided by their perceptions (Scarinzi, 2011).

This is why analyzing sports performance and experience from a solely third-person perspective only allows us to capture this action dynamic in an equation of movement and modeled behavior but does not allow us to understand how the agent experiences this environment as the one who acts. Hence, the enaction paradigm offers the possibility of articulating the different levels and domains of organization involved in sports action. As Krein and Ilundáin-Agurruza (2017) show, sport highlights the value of enactivism and extend its range of application beyond the simple minds that are usually analyzed by researchers.

Within a broader, conceptual, embodied cognition, Beilock (2008) argues that experience (i.e., practice) in/of a particular action modifies the extent to which cognition is grounded in action. Thus, embodied cognition establishes that our experience in performing a particular action helps us to predict the other’s actions in terms of what and how they will act (a similar concept to social affordances; Fajen et al., 2009). Thus, motor experience accumulated by a skilled athlete would maximize their ability to predict and accurately assess the other’s actions (Cañal-Bruland et al., 2010). However, the most prolific approach to sport is the embodied perception theory (Proffitt, 2006; Witt, 2017). This embodied viewpoint postulates that perception of the environment (e.g., ball speed) is not (solely) determined by the physical properties of the environment, but rather reflects our ability to interact with objects (Proffitt, 2006). Although this assertion may seem unaligned with the foundations of ecological psychology, embodied perception can be conceived as an extension of the concept of affordance (Gray, 2014). Hence, the properties of the environment are perceived in terms of the agent’s action capabilities (Witt and Riley, 2014; Witt et al., 2016), and within a rational boundary of possible actions to be carried out (Lessard et al., 2009), an idea which has similarities with the aforementioned affordance-based control approach (Fajen et al., 2009).

There is extensive empirical evidence of how specific perception affects the function, the relative difficulty of a task (i.e., skill expertise, concomitant success, objective task difficulty), and its final goal (overviews on Gray, 2014; Witt et al., 2016). For example, the cup was perceived to be larger by golfers who were more skilled, and when putting from nearby (Witt et al., 2008). Similar effects have been found in softball and baseball, where players with a better performance history perceived the ball to be larger (Witt and Proffitt, 2005; Gray, 2013), or when the stroke was more difficult to execute (Gray, 2013). In the same baseball study, the speed of the incoming ball was perceived as being slower by players with a better batting average (Gray, 2013). This action-specific perception of speed is claimed to be supported by an underlying perceptual-motor information process similar to calibration. By way of example, if the absolute value of ball speed is scaled to the individual agility of goalkeepers, then goalkeepers with faster lateral movement would perceive the ball as slower (Gray, 2014). With respect to the objective of the task, findings suggest that different perceptions of ball size are a function of the intended objective of the action. For example, Batters perceived a ball to be larger when the task constraints were aligned with the goal action and vice versa (Gray, 2013). Other authors have observed a correlation between hitting performance and estimated target size, but the effect disappeared when the goal of the action changed from just hitting to hitting and catching the launched ball (Cañal-Bruland and van der Kamp, 2009). Gray (2014) suggests that these differential effects of ball size perception, as a function of the goal of the intended action, may be used to shape affordance selection (e.g., altering the task constraints of the batter’s training – ball size or trajectory– to perfect a specific stroke).

In recent years, researchers have made theoretical attempts to bridge the gap between enactivism and ecological psychology (Chemero, 2009; Stapleton, 2016; Baggs and Chemero, 2018). The rapprochement between these theoretical perceptions is reflected in the transversal use of some concepts, such as affordances and agency. For example, ecological psychologists and enactivists use the term *affordances* to refer to the values or meanings of things (Thompson, 2007). Gibson himself said: “I have coined this word as a substitute for *values*, a term which carries an old burden of philosophical meaning” Gibson (1966/1968, p. 285). Even in the enactivist framework, the original concept has been changed to a broader notion called *affordance spaces* (Gallagher, 2017, p. 174). According to Travieso et al. (2020), if we are to bring enactivism closer to ecological psychology it is essential to distinguish between *perceiving* and *actualizing* affordances. These authors also comment on the relationship between affordances and sense-making, outlining that this is “because affordances are related to the bringing-forth-the-world concept of enactivism and sense-making” (p. 7).

Higueras-Herbada et al. (2019) claim that direct learning theory should be included among the post-cognitivist theories of learning, as it shares the basic commitments of embodied, embedded, enacted, and extended. According to Heras-Escribano (2019a), enactivism and ecological psychology can be combined in a single post-cognitivist research framework, providing we assume the interaction between the organic agent and the environment on two different levels of understanding. The sub-personal levels involve the neural dynamics of the sensorimotor contingencies and the emergence of *enactive* agency, and the personal level deals with the dynamics that emerge from the organism-environment interaction in *ecological* terms. It seems, then, that sensorimotor abilities and the study of affordances have much more in common than their proponents realize (Chemero, 2009, p. 154). In a more sports-related framework, the enactivism of Krein and Ilundáin-Agurruza maintains that high cognitive non-representational states during a high performance (e.g., climbing without ropes) can be possible through flow and *mushin* (i.e., mindfulness fluid awareness). The athlete is holistically attuned to the environment on multiple levels of engagement: intellectual, emotional, volitional, kinetic, and other capabilities (Krein and Ilundáin-Agurruza, 2017). Climbing is a very comprehensive sport that develops different skills in PE classes. It is interesting because the learner creates an intense relationship through a personal commitment to the wall (Terré et al., 2016). None of the learners will live the same experience. Each student must discover creative solutions that emerge moment by moment, in their constant interaction with the wall. The best hand and foot holds are not prepared in advance, but will rather, be the result of their sensorimotor enactment.

## Conclusion

In this article, we have shown the enactive and ecological notions used to explain learning in sport, PE, and daily living activities. Sports and motor skills in general are excellent settings for investigating learner cognition. Reaching a certain level of learning or mastery requires practice, and each learner experiences the learning process in a way that is unique and individual to them. We have seen how for several years, and despite having several ideas in common, enactivists and ecological psychologists have seemed to be working separately, and sometimes these ideas are at odds. However, there are clear signs of the potentials for combining the two approaches. In 2020, the beginning of a new decade, we are curious to see how events will unfold. Enactivists and neo-Gibsonians may one day no longer regard each other with suspicion, and instead join forces in a joint language, forming an *enactive-ecological* program or an *ecological-enactive* approach (cf. Baggs and Chemero, 2018; Heras-Escribano, 2019a). This would certainly allow them to broaden explanations and in our case, to better understand, how human beings function when they learn or perfect a skill. In line with the conclusions of Segundo-Ortin (2020), we believe that enactivism (Di Paolo et al., 2017; Di Paolo, 2019) can make an important contribution to understanding learning and showing how the performer acquires and optimizes his or her sensorimotor skills.

## Author Contributions

CA, JN, L-MR-P, and JZ-A have equally contributed to the structure, theoretical position, and writing of the manuscript. All authors contributed to manuscript revision, and read and approved the final version.

## Conflict of Interest

The authors declare that the research was conducted in the absence of any commercial or financial relationships that could be construed as a potential conflict of interest.

## Footnotes

1 This paper mainly uses the terms *learners* and *performers* to refer to the agents and athletes who act in all kinds of situations and who have different levels of mastery and expertise.

2 Rowlands (2010) attributed the 4E paradigm of the mind (pp. 3 and 219) to Shaun Gallagher at the Embodied Mind Workshop held at Cardiff University in July 2006 (see Gallagher, 2017, p. 28).
